# Efficient Edge-AI Models for Robust ECG Abnormality Detection on Resource-Constrained Hardware

**DOI:** 10.1007/s12265-024-10504-y

**Published:** 2024-03-12

**Authors:** Zhaojing Huang, Luis Fernando Herbozo Contreras, Wing Hang Leung, Leping Yu, Nhan Duy Truong, Armin Nikpour, Omid Kavehei

**Affiliations:** 1 School of Biomedical Engineering, The University of Sydney, NSW 2008 Sydney, Australia; 2 Department of Neurology, Royal Prince Alfred Hospital, and Central Clinical School, The University of Sydney, NSW 2006 Sydney, Australia

**Keywords:** Abnormality identification, Simple network, ECG data, Performance evaluation, Generalization, Robustness, Edge devices

## Abstract

This study introduces two models, ConvLSTM2D-liquid time-constant network (CLTC) and ConvLSTM2D-closed-form continuous-time neural network (CCfC), designed for abnormality identification using electrocardiogram (ECG) data. Trained on the Telehealth Network of Minas Gerais (TNMG) subset dataset, both models were evaluated for their performance, generalizability capacity, and resilience. They demonstrated comparable results in terms of F1 scores and AUROC values. The CCfC model achieved slightly higher accuracy, while the CLTC model showed better handling of empty channels. Remarkably, the models were successfully deployed on a resource-constrained microcontroller, proving their suitability for edge device applications. Generalization capabilities were confirmed through the evaluation on the China Physiological Signal Challenge 2018 (CPSC) dataset. The models’ efficient resource utilization, occupying 70.6% of memory and 9.4% of flash memory, makes them promising candidates for real-world healthcare applications. Overall, this research advances abnormality identification in ECG data, contributing to the progress of AI in healthcare.

**Graphical Abstract:**

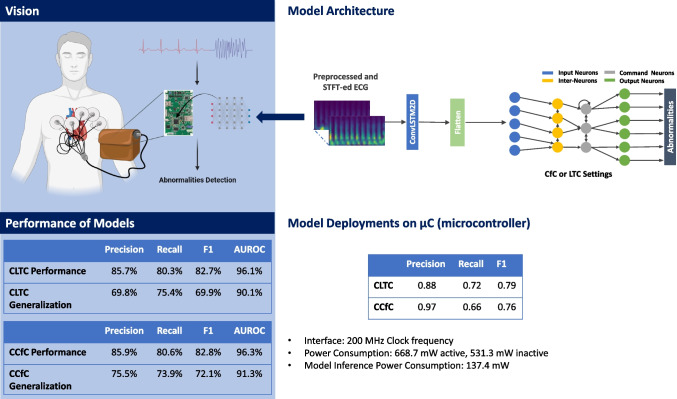

## Introduction

Various technologies have been developed to monitor heart activities, and among them, the electrocardiogram (ECG) has gained widespread use due to its non-invasiveness and cost-effectiveness. In clinical settings, the 12-lead ECG is currently considered the standard for measuring cardiac electrical activity. This technique entails positioning 12 leads, consisting of six limb leads and six chest leads, to capture heart activities from both the vertical and horizontal planes [1].

**Fig. 1:**
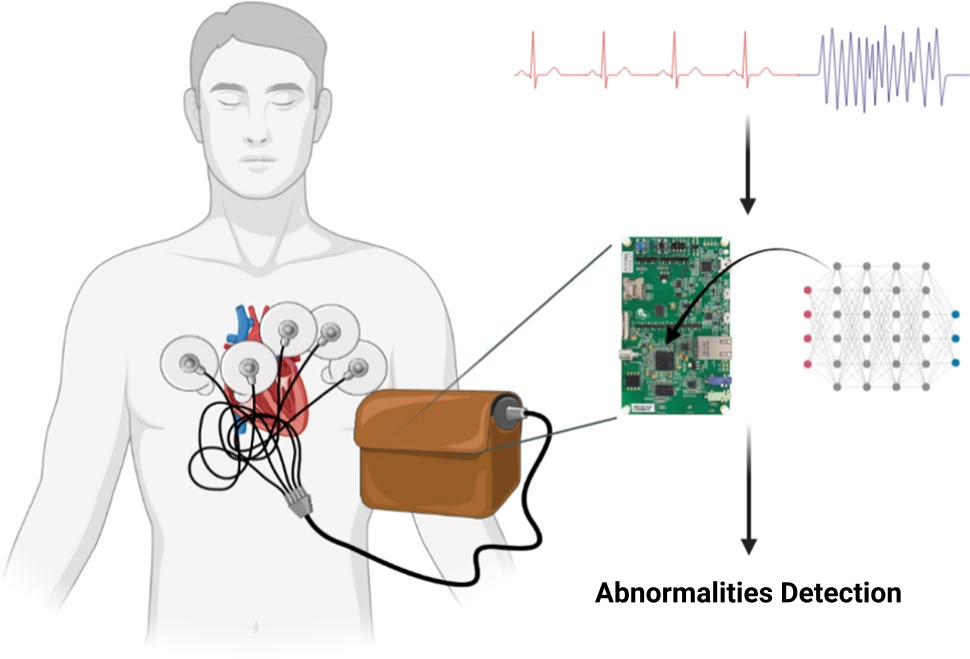
The model’s vision revolves around its application in wearable devices, where it plays a crucial role in detecting abnormalities and aiding in their identification

This study presents novel neural circuit policies (NCP) empowered models aimed at assisting clinicians in detecting the specific location where abnormality occurs. The models, as opposed to traditional recurrent neural networks such as long short-term memory (LSTM), offer the advantage of mitigating the negative effects of learning long-term dependencies on specific tasks [2]. According to the study by Mathias, the NCP model demonstrates superior computational capabilities for neurons compared to contemporary deep models [2]. Unlike common deep neural network models, which rely heavily on unpolluted input data, the NCP model exhibits higher tolerance to transient disturbances that are common in real-world conditions [2]. Additionally, the NCP model’s compact and sparse network architecture eases the interpretation process [2]. Furthermore, the model requires low memory usage, making it suitable for deployment on microcontrollers. Another advantage is that the NCP model requires only a small number of neurons. However, it has been observed that this enhancement comes at the cost of reduced accuracy performance. The current model achieved an F1 accuracy of 0.82. The vision of the work, depicted in Fig. 1, can be extended to microcontrollers, allowing its integration into wearable devices.

Indeed, the efficient resource utilization of the models is a noteworthy aspect, as they occupy a mere 240 KB, which is approximately 70.6% of the total RAM available on the STM32F746G microcontroller (equipped with 340 KB of RAM). Moreover, their utilization of approximately 96 KB of flash memory storage accounts for about 9.4% of the total flash memory available on the board, which boasts 1 MB of flash memory. This optimized use of resources renders the models highly promising candidates for real-world healthcare applications, where limitations in storage and memory capacity are critical considerations. Furthermore, the current on-chip model demonstrates reasonable power consumption, measuring at 137.4 mW. This level of power consumption is particularly crucial for ensuring that the models can operate efficiently in battery-powered devices without rapidly draining the power source. The power efficiency of these models enhances their suitability for various healthcare applications, enabling continuous and reliable operation while conserving energy resources.

### Background

In the field of abnormality detection, several notable models have been developed. Georgios proposed a hybrid CNN-LSTM network, achieving a sensitivity of 97.87% and specificity of 99.29% for classifying a specific heartbeat type using ECG data [3]. Varun Gupta introduced a model that utilized fractional wavelet transform for preprocessing, Yule-Walker Autoregressive Analysis for feature extraction, and principal component analysis for detection. This model achieved remarkable results, including a mean square error of 0.1656%, detection accuracy of 99.89%, and an output signal-to-noise ratio of 25.25 dB [4]. Tsai-Min Chen developed a model consisting of five CNN blocks, a bidirectional RNN layer, attention layer, and dense layer. This model achieved an F1 score of 0.84 in the detection of different types of ECG [5]. Jing-Shan Huang proposed a fast compression residual convolutional neural network-based model for ECG classification, which achieved an average accuracy of 98.79% [6]. [7] developed a shallow S4D model, which demonstrated a robust F1 score of 0.81 and high robustness for the input data.

For the NCP model, several notable models have also been developed. Mathias Lechner’s NCP model, originally developed for car driving applications using input from cameras, exhibits a distinct focus on the road’s horizon compared to conventional CNN models. While CNN models often prioritize roadside features and overlook the road itself, the NCP model demonstrates a different approach, placing greater emphasis on the road’s horizon [2]. This unique characteristic allows the NCP model to capture and learn global driving features effectively. This is reflected in the high variance explained by the first principal component (PC1), which reaches 92% [2]. The ability of the NCP model to concisely learn global driving features can contribute to improved understanding and decision-making in car driving applications. Ramin Hasani made advancements in liquid time-constant networks by developing closed-form continuous-time neural networks, which exhibit improved computational speed [8].

## Prerequisite

### Liquid Time-Constant (LTC) Networks

The equation 1 characterizes LTC neurons, where $\tau_{i}$ denotes the time constant of neuron *i*, determined by the ratio of its membrane capacitance $C_{m_{i}}$ to leakage conductance $g_{l_{i}}$ [2]. The activation function $\sigma _{i}(x_{j}(t))$ captures the behavior of neuron *i*, utilizing the input ${x}_{j}$ from neuron *j* and parameters $\gamma _{ij}$ and $\mu _{ij}$ to shape a sigmoid curve output between 0 and 1 [2]. The synaptic weight $w_{ij}$ reflects the strength of the connection from neuron *i* to *j*, while $x_{{leak}_{i}}$ represents the resting potential of neuron *i* [2]. Additionally, $E_{ij}$ defines the polarity of the synapse, determining its excitatory or inhibitory nature [2]. It is the building block of NCP.

1 $$\begin{aligned} \begin{aligned} \dot{x_{i}} =&- \left( \frac{1}{\tau _{i}} + \frac{\omega _{ij}}{C_{m_{i}}}\sigma _{i}({x}_{j})\right) {x}_{i} \\&+ \left( \frac{x_{leak_{i}}}{\tau _{i}} + \frac{\omega _{ij}}{C_{m_{i}}}\sigma _{i}(x_{j})E_{ij}\right) \\ { {{where\;} }\sigma _{i} (x_j(t)) = }&{\frac{1}{1 + e^{-\gamma _{ij}({x}_{j} - \mu _{ij})}}} \end{aligned} \end{aligned}$$

### Neural Circuit Policies (NCP)

The NCP model is an end-to-end learning system with several convolution layers [2]. In the NCP model, four neural layers are incorporated: sensory neurons (Ns), interneurons (Ni), command neurons (Nc), and motor neurons (Nm). Between each consecutive layer, a specific number of synapses are inserted to facilitate the flow of information [2]. Three mechanisms govern the synapse distribution: (1) synapses from source to target neurons (nso-t) following a Bernoulli distribution with probability *p*2, (2) additional synapses (mso-t) from source to target neurons without any synapses, determined by a Binomial distribution with probability *p*3, and (3) recurrent connections (lso-t) from source neurons to target command neurons, selected from a Binomial distribution with probability *p*4 [2]. The NCP model has been demonstrated to create sparse networks, enhancing interpretability compared to traditional models. Sparse networks, with fewer connections, offer a clearer and more understandable representation, facilitating improved model comprehension in various applications.

The presented equation 2 delineates the utilization of the semi-implicit Euler technique in the context of the NCP model. In this formulation, various parameters are employed to describe the dynamics of the neuronal system. Specifically, $g_{l_{i}}$ denotes the leakage conductance associated with neuron *i*, $\omega _{ij}$ signifies the synaptic weight from neuron *i* to *j*, $C_{m_{i}}$ represents the membrane capacitance of neuron *i*, $x_{leak_{i}}$ characterizes the resting potential of neuron *i*, $E_{ij}$ denotes the reversal synaptic potential governing the polarity of the synapse from neuron *i* to *j*, and $\Delta$ symbolizes the fixed step size employed in the numerical integration process.

2 $$\begin{aligned} \begin{aligned} {x}_{i}(t + \Delta ):=&\left( \frac{{x}_{i}(t)C_{{m}_{i}}}{\Delta } + g_{{l}_{i}}x_{ {leak}i} \right. \\&+ \left. \sum {j \in I_{ {in}}} \omega _{ij}\sigma _{i}(x_j(t))E_{ij}\right) \\&/ \left( \frac{C_{m_i}}{\Delta } + g_{l_i} + \sum _{j \in I_{ {in}}} \omega _{ij}\sigma _{i}(x_j(t))\right) \end{aligned} \end{aligned}$$

### Closed-Form Continuous-Time (CfC) Neural Networks

The closed-form solution for the interaction between neurons and synapses in continuous-time neural networks offers a substantial advantage by significantly improving efficiency, making training and inference between one and five orders of magnitude faster compared to models relying on numerical differential equation solvers [9]. Additionally, the CfC derived from liquid time-constant dynamics demonstrates notable scalability and performance in time-series modeling, showcasing their suitability for a wide range of applications [9]. The CfC model consists of an input perception module, LTC module, and outputs [9]. A notable characteristic of closed-form control (CfC) neural networks is that they do not rely on numerical ordinary differential equation (ODE) solvers to generate their temporal rollouts [9]. This kind of network not only achieves the flexible, causal, and continuous-time feature of ODE-based networks but also has better efficiency compared to them [9]. The CfC model can be represented by the equation 3, where $\sigma (-f(x,I;\theta _{f})t)$ and $[1 - \sigma (-[f(x,I;\theta _{f})]t)]$ are the time-continuous gating, and $g(x,I;\theta _{g})$ is an independent network compartment [9].

3 $$\begin{aligned} \begin{aligned} X(t) =&\sigma (-f(x,I;\theta _{f})t) \odot g(x,I;\theta _{g}) \\&+ [1 - \sigma (-[f(x,I;\theta _{f})]t)] \odot h(x,I;\theta _{h}) \end{aligned} \end{aligned}$$

## Datasets

In this study, the proposed models were evaluated using two distinct datasets. The first dataset, referred to as the CPSC dataset or the 12-lead ECG dataset, was created for The China Physiological Signal Challenge 2018 [10]. Its purpose was to facilitate the automatic detection of abnormalities in rhythm and morphology within 12-lead ECGs. The second dataset employed in this research is the Telehealth Network of Minas Gerais (TNMG) dataset [11], primarily used for model training.

To assess the models’ performance on new and unseen data, the CPSC dataset was utilized as an independent test dataset. This evaluation aimed to measure the models’ ability to handle real-world data that differs from the TNMG dataset.

The study’s objective was to evaluate the models’ generalization and performance on unfamiliar data by training them on the TNMG dataset and evaluating their effectiveness on the CPSC dataset. This evaluation is crucial for determining the models’ reliability and efficacy in real-life scenarios.

### TNMG

The TNMG dataset employed in this study consists of uniquely different 2,322,513 labeled samples, each representing 10 seconds of 12-lead electrocardiogram (ECG) data. These samples represent six distinct types of abnormalities: atrial fibrillation (AF), first-degree atrioventricular block (1dAVb), left bundle branch block (LBBB), right bundle branch block (RBBB), sinus bradycardia (SB), and sinus tachycardia (ST) [11]. The ECG data was originally sampled at 400 Hz frequency.

To create a balanced dataset for model training, 3000 data were selected for each of the six abnormalities at random, along with a further 3000 data without any abnormalities. This resulted in a total sampled dataset size of 21,000. In cases where patients exhibited multiple abnormalities, any remaining samples needed to achieve the subset size of 21,000 were chosen from the TNMG at random. For more detailed information about these six abnormalities, please refer to Table 1.

**Table 1:** Abnormalities within the TNMG dataset are categorized into different classifications

Abnormality	Description
Atrial fibrillation (AF)	It is the most prevalent form of arrhythmia, displaying symptoms of a fast and irregular heartbeat [12]
Sinus bradycardia (SB)	This condition is characterized by a slower firing of electrical impulses from the heart’s sinus node, resulting in a heart rate that is slower than the typical resting rate [13]
Sinus tachycardia (ST)	This condition is characterized by an increased resting heart rate and an exaggerated heart rate response to mild physical exertion or changes in body posture, indicating a tachyarrhythmia [14]
Right bundle branch block (RBBB)	The condition disrupts the normal electrical activity in the heart’s ventricles, leading to a delay in the depolarization of the right ventricle. This delay is caused by interrupted signal transmission in the His-Purkinje system [15]
Left bundle branch block (LBBB)	As a consequence, there is a specific order of activation in the right ventricle preceding the left ventricle, resulting in subsequent changes in perfusion, mechanics, and workload within the left ventricle [16]
First-degree atrioventricular block (1dAVb)	When a surface electrocardiogram reveals a PR interval that exceeds 200 ms, it is indicative of a first-degree atrioventricular block [17]

**Fig. 2:**
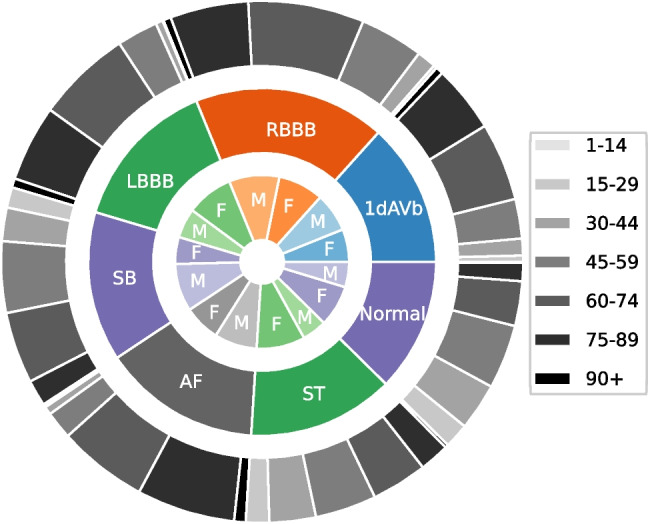
The presented TNMG subset represents a highly balanced dataset containing six types of abnormalities. Additionally, it exhibits a higher concentration of older patients, which is reflective of the general population

The dataset underwent a process of normalization, where it was adjusted to a consistent length of 4096 readings, ensuring uniformity and facilitating analysis and modeling. Any readings exceeding this length were removed, streamlining data processing and comparison. Figure 2 illustrates a balanced distribution of genders in the resampled dataset, promoting inclusivity and valid analysis. The dataset also reflects the age distribution observed in the general population, enhancing representativeness for age-related analysis. Additionally, the sampling method yielded a balanced distribution of different abnormalities, allowing a comprehensive evaluation of their characteristics and impacts. This balanced dataset improves the model’s learning process and overall performance [18].

### CPSC

The CPSC dataset comprises distinct 12-lead electrocardiograms (ECGs) ranging from 6 to 60 seconds, each recorded at a sample rate of 500 Hz. To ensure compatibility with the CPSC dataset, the TNMG data was resampled at a rate of 500 Hz specifically for training purposes. This dataset is notable due to its inclusion of electrocardiograms (ECGs) from patients who have been diagnosed with a range of cardiovascular conditions and exhibit common rhythms. The ECGs in the dataset have been expertly labeled, providing accurate annotations for these abnormalities. Overall, the dataset encompasses eight distinct types of abnormalities.

To effectively test the model’s generalization, we conducted tests using four selected abnormalities from the dataset: atrial fibrillation (AF), left bundle branch block (LBBB), right bundle branch block (RBBB), and first-degree atrioventricular block (1dAVb). However, it is important to note that this study does not include four other types of abnormalities: premature atrial contraction (PAC), premature ventricular contraction (PVC), ST-segment depression (STD), and ST-segment elevated (STE).

**Fig. 3:**
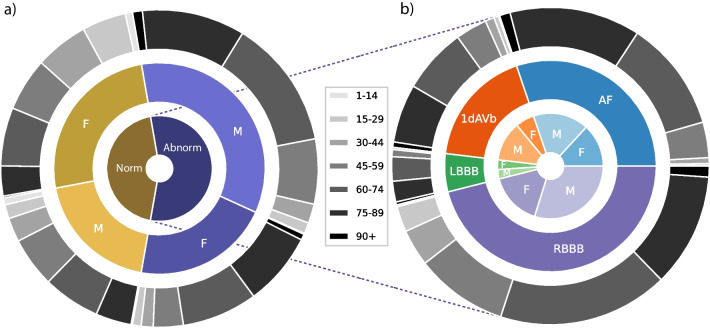
**a** The distribution of patients with studied abnormalities versus those without studied abnormalities or with normal ECG readings. **b** A detailed analysis of patients with studied abnormalities, breaking down the data into specific individual abnormalities

As part of the data selection process, any entries in the dataset with missing readings were excluded, resulting in a final dataset of 6877 distinct ECG tracings. The data was subsequently standardized by adjusting it to a consistent length of 4096 readings. Any additional readings beyond this length were removed from the dataset during the cleaning process. For more detailed information about the refined dataset, please refer to Fig. 3.

The analysis of the dataset uncovered a gender disparity, indicating that there is a greater representation of male patients compared to female patients. However, the age distribution of the patients in the dataset aligns with that of the general population, with a larger proportion of individuals belonging to older age groups. However, when examining the distribution of abnormalities, a slight imbalance is observed. Specifically, the occurrence of LBBB is relatively lower compared to the prevalence of other abnormalities present in the dataset.

## Methods

Our primary objective is to create a model that is compact, efficient, and well-suited for processing electrocardiogram (ECG) data. We aim to optimize the model specifically for hardware implementation, ensuring its compatibility with resource-constrained devices. Furthermore, we conduct a comprehensive evaluation of the model’s performance in terms of both generalization and robustness to incomplete data.

To achieve our goal, we developed a small-scale architecture that minimizes computational requirements without compromising accuracy. The ECG data is subjected to essential preprocessing steps, such as filtering and short-time Fourier transform (STFT) transformation, to enhance its quality and extract relevant features.

Afterwards, the preprocessed data is inputted into the proposed models for the purposes of training and validation. The performance of the model is then assessed using selected performance metrics in order to gain insights into its effectiveness.

### Preprocessing of ECG Data

To reduce noise interference in the ECG signal, a Butterworth band-pass filter is applied. The Butterworth filter is selected for its uniform response to all desired frequencies [19]. The passband of the band-pass filter is set from 0.5 to 40 Hz. This frequency range is chosen to retain important information such as the T wave, P wave, and QRS complex, while effectively removing powerline noise at 50 Hz [20].

After the signal is filtered, the short-time Fourier transform (STFT) is applied. Unlike the fast Fourier transform (FFT), which operates on the entire signal at once, the STFT divides the signal into smaller windows and applies the FFT to each of these windows individually. This approach allows for the extraction of both frequency and time-related information from the analysis. By analyzing the signal in smaller time segments, the STFT captures changes in the signal over time, providing a more detailed representation of the signal’s time-varying frequency components. This enables the gathering of both frequency and temporal information from the signal analysis. The equation for FFT is shown in Eq. 4 where *X*[k] represents complex spectrum at frequency index *k*, *x*[*n*] represents the input sequence of length *N*, and $e^{-j2\pi kn/N}$ represents the complex exponential at frequency index *k* and time index *n*. The formula for STFT is shown in Eq. 5, where $X(t, \omega )$ represents the complex value of the STFT at time *t*, frequency $\omega$, $x(\tau )$ represents the input signal, and $w(\tau - t)$ denotes the window function centered at time *t*.

4 $$\begin{aligned} X[k] = \sum _{n=0}^{N-1} x[n] \cdot e^{-j2\pi kn/N} \end{aligned}$$

5 $$\begin{aligned} X(t, \omega ) = \int _{-\infty }^{\infty } x(\tau ) \cdot w(\tau - t) \cdot e^{-j\omega \tau } \, d\tau \end{aligned}$$

### Neural Circuit Policies (NCP)

Inspired by the Caenorhabditis elegans nematode, the neural circuit policies (NCPs) have been developed as brain-inspired intelligent agents. Unlike contemporary deep models, each neuron within the NCP framework exhibits heightened computational capabilities. Through extensive research, it has been demonstrated that the adoption of NCPs leads to the creation of sparse networks, which, in turn, offer enhanced interpretability compared to conventional models [2].

A NCP network consists of a collection of liquid time-constant (LTC) neurons [8]. By implementing the aforementioned NCP design principles, the outcome is highly condensed and sparsely interconnected networks of LTC neurons. LTC neurons in the simulation are commonly represented as leaky integrators, where they accumulate incoming inputs over time and gradually release a portion of the accumulated charge. This leakage mechanism is vital for avoiding saturation of the neuron’s membrane potential and facilitating the processing of temporal information [2].

In addition, this study introduces an independent model for closed-form continuous-time neural networks (CfC) in the context of time-series modeling. Derived from liquid networks, CfC models outperform advanced recurrent neural networks. They employ closed-form ordinary differential equations (ODEs) and approximate the solution for a previously unsolved integral in liquid time-constant dynamics [9].

**Fig. 4:**
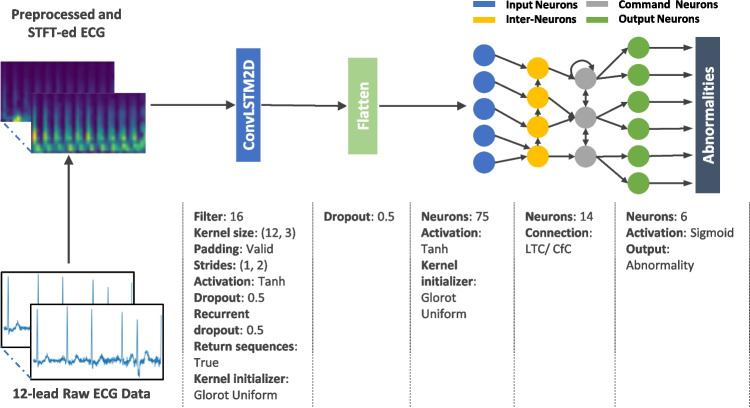
The model architecture consists of a ConvLSTM2D layer that handles a preprocessed ECG data after undergoing STFT transformation. The ConvLSTM2D layer extracts features, which are then densely connected to 75 neurons, serving as the input neurons for the NCP network. The input, motor, and output neurons are interconnected using either LTC or CfC arrangements, with a sigmoid activation function applied at the final stage

### Simple Model Architecture

The proposed model architecture, illustrated in Fig. 4, is designed to be simple yet effective. It consists of a single ConvLSTM2D layer responsible for feature extraction, connected to 75 neurons serving as the input neurons for the NCP network. The network can be constructed using either the LTC or CfC arrangement, resulting in two distinct models: CLTC and CCfC models with 14 inter and command neurons and 6 output neurons.

The design rationale was crafted to suit the constraints inherent in microcontroller environments, leading to the pursuit of a carefully streamlined architecture featuring a single layer of ConvLSTM2D. Additionally, to optimize efficiency within the NCP framework (CLTC and CCfC), a conscious decision was made to limit the network to a modest configuration of 20 neurons, consistent with the structure proposed by [2]. The determination of the most efficient hyperparameters evolved through a systematic exploration, involving a methodical examination of diverse hyperparameter combinations across a series of experiments. The outcomes of this empirical investigation are visually depicted in Fig. 4.

### Evaluation Metrics

Precision 7 and recall 6 are metrics used to evaluate the performance of a model, with precision measuring the accuracy of positive predictions and recall capturing the proportion of actual positives correctly identified. The F1 score 8, is the harmonic mean of precision and recall, balancing the precision-recall trade-off.

6 $$\begin{aligned} {Recall} = \frac{TP}{FN + TP} \end{aligned}$$

7 $$\begin{aligned} {Precision} = \frac{TP}{FP + TP} \end{aligned}$$

8 $$\begin{aligned} F_{1} = 2 \times \frac{ {Recall} \times {Precision}}{ {Recall} + {Precision}} \end{aligned}$$

The area under the receiver operating characteristic curve (AUROC) is a performance metric employed to evaluate the discriminative capacity of a model in distinguishing between negative and positive cases at different threshold values. It quantifies the model’s ability to classify instances correctly and is commonly employed in binary classification tasks.

These metrics, such as F1 score, precision, recall, and AUROC, are widely used to evaluate the performance of machine learning models in binary classification tasks, particularly in abnormality detection.

## Experiment

### Training

In the experimental phase, the preprocessed TNMG subset data is employed as the training dataset, where training and validation procedures are carried out on both the CLTC and CCfC models. To address the class imbalance issue in multi-labeled datasets, a strategy was employed where data instances with positive labels were duplicated twice. This replication technique was implemented to ensure an adequate number of positive-labeled data points within the training dataset, helping to balance the distribution between positive and negative labels. The replication process was exclusively applied to the training data, with no application to the validation and test datasets. Furthermore, the trained models are deployed onto a microcontroller to showcase their feasibility for chip deployment.

The models were trained on Intel(R) Xeon(R) CPU E5-2630 v3 @ 2.40GHz. During the training process, a batch size of 128 was used, and the models underwent 300 epochs of training. The learning rate was set to 0.01, and the Adam optimizer was employed with the binary cross-entropy loss function.

The preprocessed CPSC dataset is utilized to assess the generalization capabilities of the models. Additionally, the robustness of the models is evaluated by testing their performance when exposed to corrupted data inputs.

### In-sample Training and Validation

To evaluate the performance of the models, a dedicated subset comprising 20% of the TNMG data was set aside for validation purposes. The specific data points used for evaluation were deliberately withheld from the model’s training process and reserved specifically for assessing the model’s performance. This validation process was conducted independently for each model, enabling a direct comparison of their respective performances.

### Model Deployment

After training, the models are deployed onto a microcontroller, and the validation data is utilized to perform inference on the microcontroller. This enables the observation of the deployed models’ performance in a real-world setting, specifically on the microcontroller platform.

### Test on Unseen Data

The performance of the models will be thoroughly assessed on the CPSC dataset, which is carefully selected to reflect real-world scenarios. By comparing the models’ predictions with known values using performance metrics, their strengths and limitations will be evaluated. The findings will guide decisions regarding the effectiveness of the models and suggest potential enhancements for better ECG analysis.

### Model Robustness

The models are subjected to rigorous robustness testing, involving the introduction of white noise and the systematic removal of individual channels from the 12-lead ECG data. This comprehensive evaluation aims to gain insights into the models’ performance under varying conditions. Additionally, channels are deliberately and randomly omitted from the 12-lead ECG data to further challenge the models in handling missing inputs.

The removal of channels occurs in a progressive manner, ranging from 1 to 6 leads, allowing for a nuanced examination of the models’ accuracy across different scenarios. Performance metrics will be meticulously applied to gauge and compare the models’ effectiveness in each test. These metrics serve as crucial benchmarks, highlighting areas where the models excel and pinpointing potential areas for improvement. The findings from these assessments will offer valuable insights, guiding potential enhancements to optimize the models’ robustness.

## Result

This section will provide a comprehensive analysis of the models’ performance. Moreover, the discussion will also include an in-depth exploration of their generalizability and robustness.

### Performance of the Models

**Fig. 5:**
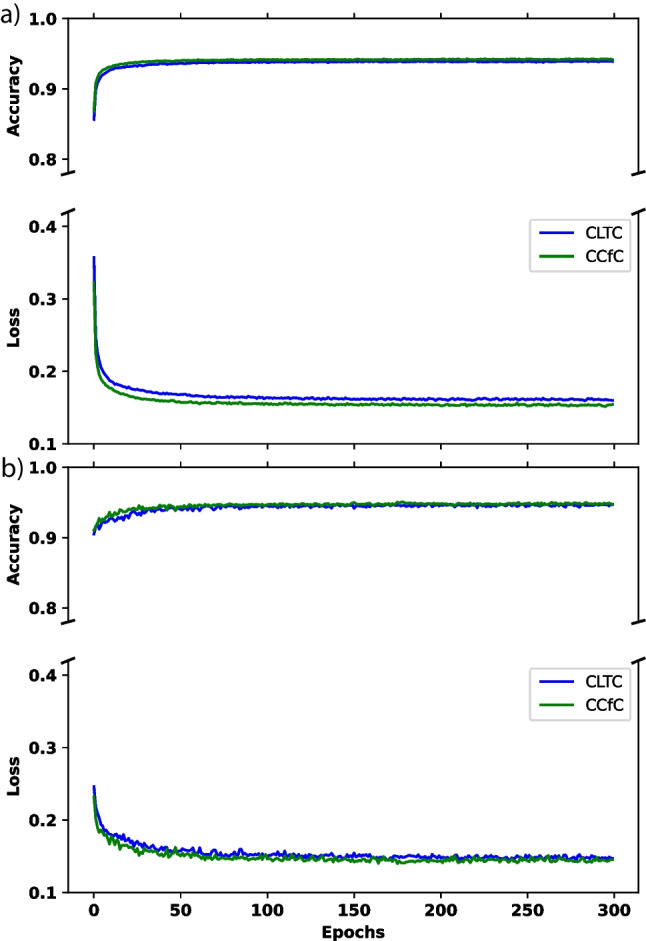
**a** The performance of the two models during the training process. **b** The performance of loss and accuracy metrics in validation

This research study primarily revolves around training the proposed models using the TNMG subset dataset, as previously mentioned in the paper. The TNMG subset dataset serves as the main training data for developing and evaluating the models in this study. The training process was conducted over 300 epochs, with careful monitoring and recording of metrics such as accuracy and loss for both the validation and training sets at each epoch. The figures presented in the paper visually illustrate the recorded metrics, providing a comprehensive overview of the entire training procedure. Notably, the performances of both the CLTC and CCfC models were assessed using identical settings, and their individual performances are plotted in Fig 5.

Figure 5a demonstrates a consistent decrease in training loss as the training progresses, indicating that the model’s performance improves over time. Simultaneously, the training accuracy steadily increases and eventually reaches a stable level, indicating that the model successfully learns from the training data. Although both models have achieved similar results, the CCfC model has slightly higher accuracy than the CLTC model.

Throughout the training process, a general upward trend is observed in the validation accuracy, indicating an improvement in the model’s performance on unseen data. Additionally, the validation loss consistently decreases over time, suggesting that the model’s predictions align more closely with the ground truth labels during validation. Additionally, Fig. 5b illustrates that the two models behave similarly, but the CCfC model continues to outperform the CLTC model slightly.

**Table 2:** CLTC model validation results (*P* precision, *R* recall)

Class	P	R	F1	AUROC
1dAVb	71.2%	52.7%	60.6%	88.1%
RBBB	92.5%	84.0%	88.1%	97.2%
LBBB	91.2%	93.6%	92.4%	99.1%
SB	89.3%	92.6%	90.9%	99.0%
AF	77.5%	70.5%	73.8%	94.1%
ST	92.5%	88.7%	90.6%	99.3%
Average	85.7%	80.3%	82.7%	96.1%

**Table 3:** CCfC model validation results (*P* precision, *R* recall)

Class	P	R	F1	AUROC
1dAVb	75.9%	52.2%	61.8%	89.3%
RBBB	92.3%	82.9%	87.3%	97.1%
LBBB	88.9%	93.2%	91.0%	99.1%
SB	89.2%	92.9%	91.0%	99.0%
AF	78.7%	71.3%	74.8%	93.7%
ST	90.4%	91.5%	90.9%	99.3%
Average	85.9%	80.6%	82.8%	96.3%

The performance of the CLTC and CCfC models is summarized in Tables 2 and 3, respectively. Both models have achieved comparable performance, with an F1 score of 0.827 for CLTC and 0.828 for CCfC and AUROC values of 0.961 for CLTC and 0.963 for CCfC. These performance metrics align with the trends observed in Fig. 5.

In addition to differences in performance and accuracy, another notable observation between the two models is their contrasting training speeds. The CCfC model exhibits faster training times (approximately 88 s/epoch) in contrast to the CLTC model (approximately 92 s/epoch). This discrepancy is attributed to the CfC being an approximation of LTC, designed to streamline computational efficiency for faster processing. It is worth noting that both models were trained on CPUs instead of GPUs. This observation suggests that the simple model architecture requires fewer calculations and less computational power during training.

### Model Deployments on Micro-controller

This experiment successfully deployed CLTC and CCfC models on an STM32F746G Discovery board, a resource-constrained edge device featuring an STM32F746NGH6 Arm Cortex core-based microcontroller. With its 1 Mb of flash memory and 340 Kbytes of RAM, this board proved capable of managing the complex computational tasks required for neural network inference. The software solution STM32Cube.AI, developed by STMicroelectronics, was instrumental in optimizing the conversion and deployment of the pretrained models for the STM32 microcontrollers. The preprocessed data was saved on the microcontroller, ready to be input into the pretrained model.

Both models were compared to the previous batch of 30 inferences executed on the STM32F746G Discovery board. It reported a precision of 0.88, and the recall was approximately 0.72 for CLTC. The F1 score, a measure that balances precision and recall, was approximately 0.79, indicating well-rounded model performance.

Similarly, the CCfC model also displayed notable performance. The model reported a commendable precision of 0.97. However, the recall was about 0.66, slightly lower than the CLTC model. The F1 score was approximately 0.76, illustrating a fairly balanced model performance regarding precision and recall.

The measurement of power consumption was conducted using the STM32 Power Shield, a Nucleo expansion board. When the interface runs at a clock frequency of 200 MHz, the power consumption is measured at 668.7 mW. On the other hand, when the interface is not running, the power consumption is observed to be 531.3 mW. As a result, the model itself consumes approximately 137.4 mW.

The inference time for 10 seconds of preprocessed ECG data on the STM32F746G Discovery board was measured at approximately 1 second. The utilization of a dedicated digital signal processing unit for preprocessing the signal during the inference process enhances the feasibility of achieving real-time processing in scenarios that demand immediate model outputs.

These results represent a significant milestone for machine learning in edge devices, showcasing the viability of deploying sophisticated deep learning models on resource-constrained hardware. However, there remains ample room for improvement. Future work will explore the potential of deploying the models using TinyEngine, a compact inference library developed by MIT researchers [21, 22]. This platform may enhance the accuracy of the neural network model when inference is performed on edge devices, further advancing AI on edge devices [21, 22]. The findings of this experiment highlight the feasibility and potential of deploying deep learning models in such environments, opening a new frontier for AI applications.

### Generalization of Models

Predictions were made on the CPSC dataset using the trained models on the TNMG subset, as described in the data section, to assess the generalization ability. This evaluation aimed to measure how effectively the models could handle new and unseen data from the CPSC dataset, which differs from the training dataset. It is important to mention that the CPSC dataset contains eight different types of abnormalities, but only four of them are also present in the TNMG dataset. By evaluating the chosen model’s performance on this subset, we can gather valuable information about its capacity to apply learned knowledge to new and unseen data.

**Table 4:** CLTC model generalization results (*P* precision, *R* recall)

Class	P	R	F1	AUROC
1dAVb	47.8%	65.2%	55.2%	86.7%
RBBB	91.5%	60.8%	73.0%	83.8%
LBBB	89.0%	82.6%	85.7%	96.6%
AF	50.8%	92.9%	65.7%	93.4%
Average	69.8%	75.4%	69.9%	90.1%

**Table 5:** CCfC model generalization results (*P* precision, *R* recall)

Class	P	R	F1	AUROC
1dAVb	72.9%	57.9%	64.6%	88.9%
RBBB	90.4%	61.0%	72.9%	84.7%
LBBB	88.2%	82.6%	85.3%	97.2%
AF	50.6%	94.1%	65.8%	94.4%
Average	75.5%	73.9%	72.1%	91.3%

The performance of the two models on unseen data is presented in Tables 4 and 5, demonstrating their ability to perform well. The CLTC model achieves an average F1 score of 0.70 and an AUROC of 0.90, while the CCfC model achieves an F1 score of 0.72 and an AUROC of 0.91. These results show that both models possess strong generalization capabilities, with the CCfC model exhibiting slightly better generalization performance than the CLTC model.

While the dataset owners haven’t explicitly disclosed specific differences, the possibility of disparities affecting the model’s generalizability cannot be overlooked. The TNMG and CPSC datasets demonstrate potential variations in the number of bits per channel. Notably, the TNMG dataset may utilize a unique bit depth, distinct from the configuration employed in the CPSC dataset. Variations in filter settings may also be present within the datasets due to preprocessing by the dataset owners. Differences in data treatment could potentially influence the model’s generalizability. Despite incorporating filters during the training process, complete elimination of all disparities between the two datasets may not be guaranteed. Additionally, disparities in the sampling rates of the two datasets were identified. To address this dissimilarity, data resampling techniques were strategically employed to align sampling rates across both datasets. This precautionary step was taken to proactively mitigate potential impacts on the model’s generalizability, ensuring a more consistent and effective learning experience.

### Model Robustness

**Fig. 6:**
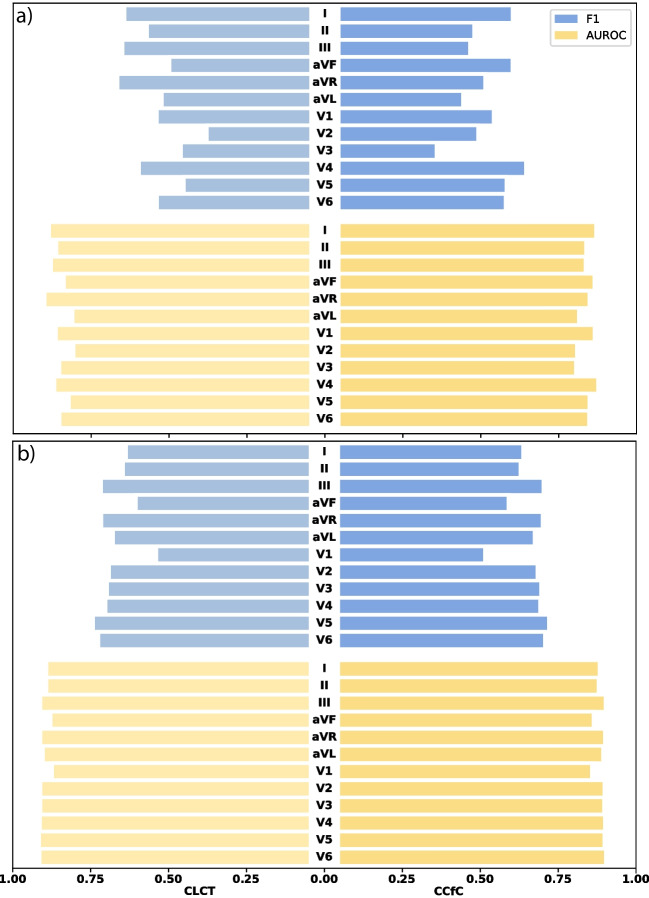
**a** An analysis of the model’s performance (F1 and AUROC) when subjected to white noise with a standard deviation of 0.1 introduced in each channel. **b** A visualization of the model’s performance (F1 and AUROC) in response to the intentional blanking out of each channel. The left side corresponds to the CLTC, while the right side represents the CCfC model

In our pursuit of assessing the models’ robustness, comprehensive tests were conducted to evaluate their performance under diverse conditions. Firstly, white noise was deliberately introduced to each of the 12 channels of the CPSC dataset, enabling us to gauge the models’ resilience and efficacy in the presence of such disturbances. Additionally, a separate evaluation involved the intentional removal of information from each of the 12 lead ECG channels, allowing us to meticulously analyze the models’ performance when confronted with data deficiencies. These tests collectively contribute to a thorough understanding of the models’ robustness across varying scenarios. The outcomes of these rigorous tests are succinctly encapsulated in Fig. 6.

It is evident that white noise exerts a more pronounced impact on the models’ performance, particularly concerning the F1 score, as compared to the effects induced by blanked-out channels. Additionally, variations in the impact are discernible across different channels. Furthermore, it is noteworthy that the CCfC model exhibits greater robustness compared to the CLTC model in navigating both the challenges posed by noise and the blanked-out channel tests. This observation underscores the superior resilience of the CCfC model across various stressors, contributing to its commendable performance under adverse conditions. Despite the models demonstrating enhanced robustness in handling empty channels, a more comprehensive examination was conducted by subjecting the model to multi-channel blanking to assess the extent of its limitations.

To further assess the model’s performance, we conducted additional evaluations on the CPSC dataset by randomly removing multiple channels from the 12-lead ECG data. This allowed us to investigate the model’s robustness and capability to handle incomplete or missing input information.

**Fig. 7:**
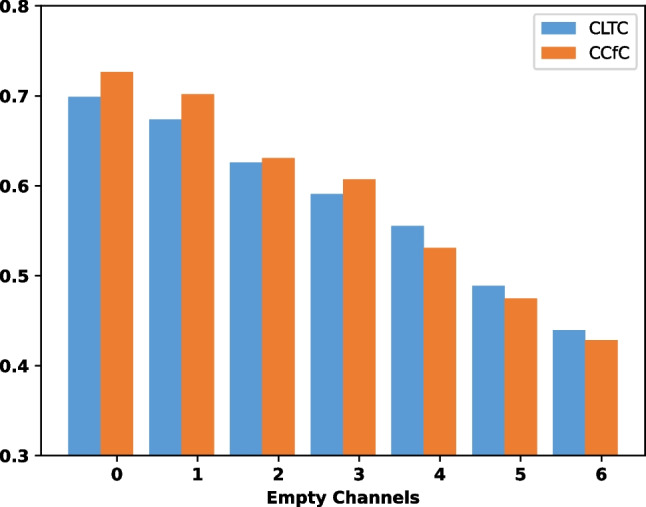
The F1 metrics of the models were evaluated based on the number of empty channels in the 12-lead CPSC ECG data

In Fig. 7, it can be observed that as the number of emptied leads increases, the F1 performance metric of the models declines. Initially, the CCfC model demonstrates superior performance compared to the CLTC model. However, an interesting finding emerges as the number of empty channels increases: the CLTC model starts to outperform the CCfC model. This suggests that the CLTC model may exhibit better robustness when faced with more empty channels.

## Discussion

The results provide a comprehensive analysis of the models’ performance, their generalizability, and robustness.

Regarding the performance of the models, both the CLTC and CCfC models achieved similar results in terms of F1 scores and AUROC values. The CCfC model exhibits proficient handling of both noise and empty channels when applied to individual channels. The CCfC model demonstrated slightly higher accuracy compared to the CLTC model but with an increasing number of empty channels, the CLTC model showed a higher accuracy, which means the CLTC model had a better robustness when faced with a higher number of empty channels compared to the CCfC model. The training process showed that the models consistently improved their performance during training, with decreasing loss and increasing accuracy. The validation results also indicated that both models performed well, with the CCfC model slightly outperforming the CLTC model.

The models were successfully deployed on a resource-constrained microcontroller, the STM32F746G Discovery board, demonstrating the feasibility of running complex deep learning models on edge devices. The deployed models achieved commendable precision and recall, resulting in the CCfC model exhibiting faster training speed than the CLTC model.

The generalization performance of the models was evaluated on the CPSC dataset, which included abnormalities not present in the training data. Both models demonstrated good generalization capabilities, with the CCfC model showing slightly better performance regarding F1 scores and AUROC values.

**Table 6:** Comparison with state-of-the-art works in the field

References	Models	F1
Mukhopadhyay et al. [23]	Deep Q-learning NAS framework	0.83
Chen et al. [24]	Multi-layer perceptron (MLP)	> 0.8
Our work	CLTC and CCfC	0.83

Table 6 presents a comparative analysis of our work alongside other contributions in the field. In the investigation led by [23], they utilized an advanced deep Q-learning NAS framework, yielding an impressive F1 score of 0.83 for atrial fibrillation detection on a microcontroller. Similarly, [24] employed a multi-layer perceptron, achieving a commendable F1 score surpassing 0.8 in their atrial fibrillation detection model on a microcontroller. While these models exhibit performance comparable to ours, it is essential to underscore that both are confined to single-class classification, setting them apart from our current work. Our research surpasses these constraints by extending its capabilities to effectively handle multiple classes.

Overall, the results demonstrate the effectiveness and potential of the proposed models for abnormality identification. The models showed promising performance, generalization capabilities, and the ability to handle incomplete or missing input information. Further improvements can be explored, such as deploying the models with optimized inference libraries for edge devices, which may enhance their accuracy in resource-constrained environments.

## Limitations

While the CLTC and CCfC models exhibit promising results in abnormality identification using ECG data, certain limitations should be acknowledged:

- **Scalability concerns**: The proposed models were primarily designed and evaluated for resource-constrained microcontroller environments. While successful in these settings, their scalability to larger computational platforms or diverse hardware configurations remains unexplored. Future research may investigate adaptations or optimizations to ensure efficient performance across a broader range of computing resources.
- **Interpretability challenges**: The simplicity of the proposed model architecture, especially the single recurrent neural network (RNN) layer in the form of ConvLSTM2D, connected to a limited number of neurons, enhances efficiency but may compromise interpretability. RNN architectures, such as ConvLSTM2D, inherently pose challenges for interpretability. Understanding the decisions of complex deep learning models is particularly challenging, and further efforts may be required to enhance transparency, especially in healthcare applications where comprehending model decisions is crucial.
- **Limited exploration of hyperparameter space**: The determination of hyperparameters, while conducted systematically, may not cover the entirety of the hyperparameter space. A more exhaustive exploration could provide additional insights into optimal configurations for diverse datasets or different edge device specifications.
- **Focused evaluation**: The evaluation primarily emphasizes abnormality identification in ECG data, and the models’ generalizability is demonstrated on the CPSC dataset. However, a more comprehensive evaluation across a wider array of diverse healthcare datasets could provide a more nuanced understanding of the models’ performance across various clinical scenarios.
- **Optimization opportunities for edge devices**: Although the models were deployed on a resource-constrained microcontroller, further exploration into deploying the models with optimized inference libraries for edge devices could enhance their accuracy and efficiency in real-world, resource-constrained healthcare environments.
- **Incomplete exploration of edge device constraints**: While the models were successfully deployed on the STM32F746G Discovery board, the study does not extensively explore various edge device constraints or potential challenges that may arise in real-world deployment scenarios. Future research could delve deeper into the adaptability of the models to different edge devices with varying capabilities.

Acknowledging these limitations, the CLTC and CCfC models present a valuable contribution to abnormality identification in ECG data, laying the groundwork for further advancements in scalable and interpretable deep learning models for healthcare applications.

## Conclusion

In conclusion, using ECG data, this study demonstrated two models, CLTC and CCfC, for abnormality identification. Both models demonstrated comparable performance and good generalization capabilities on unseen data. The models were successfully deployed on a resource-constrained microcontroller, showcasing their potential for edge device applications. The findings highlight the effectiveness of the models in abnormality detection and their ability to handle incomplete input. Further improvements can be explored, such as optimizing inference libraries and expanding the training data. Overall, these models contribute to the advancement of AI in healthcare and hold promise for the early detection and treatment of cardiac conditions.

## Clinical Relevance

This study introduces CLTC and CCfC models to enhance ECG abnormality identification in real-world clinical settings, catering to specific clinical needs and demonstrating comparable performance metrics.

In practical healthcare applications, the choice between real-time classification and off-line evaluation depends on the clinical use case. Immediate decision-making, as in emergencies, benefits from real-time classification. Conversely, scenarios like retrospective analyses or continuous monitoring align with off-line evaluation.

The adaptability of these models to resource-constrained microcontrollers makes them promising for edge device deployment, addressing concerns of data privacy, latency, and network connectivity in real-world clinical settings. For instance, processing ECG data on the edge allows timely abnormality identification without continuous cloud connectivity, ideal for remote or point-of-care settings.

Validation on the CPSC dataset highlights the models’ versatile application in various clinical contexts. Their efficient resource utilization positions them as potential contributors to improving clinical diagnostics and patient care, especially in settings with limited computational resources. This research aims to integrate advanced AI technologies, contributing to the ongoing evolution of healthcare practices by addressing specific needs and constraints in real-world clinical scenarios.

## Data Availability

This research paper utilizes the publicly accessible CPSC dataset for analysis and experimentation. However, it is crucial to acknowledge that the TNMG dataset, on the other hand, is not publicly available. To gain access to the TNMG dataset, permission must be obtained from the data owner.

## Code Availability

The code is available through the following link: https://github.com/NeuroSyd/NCP. Please note that certain terms, conditions, or usage restrictions may apply to the code.

## References

[1] Meek S, Morris F. ABC of clinical electrocardiography: Introduction. I-Leads, rate, rhythm, and cardiac axis. BMJ: British Medical Journal. 2002;324(7334):415. 11850377 10.1136/bmj.324.7334.415 PMC1122339

[2] Lechner M, Hasani R, Amini A, Henzinger TA, Rus D, Grosu R. Neural circuit policies enabling auditable autonomy. Nat Mach Intell. 2020;2(10):642–52. 10.1038/s42256-020-00237-3

[3] Petmezas G, Haris K, Stefanopoulos L, Kilintzis V, Tzavelis A, Rogers JA, Katsaggelos AK, Maglaveras N. Automated atrial fibrillation detection using a hybrid CNN-LSTM network on imbalanced ECG datasets. Biomedical Signal Processing and Control. 2021;63: 102194. 10.1016/j.bspc.2020.102194

[4] Gupta V, Mittal M, Mittal V. A novel FrWT based arrhythmia detection in ECG signal using YWARA and PCA. Wireless Personal Communications. 2022, pages 1–18.

[5] Chen T-M, Huang C-H, Shih ESC, Hu Y-F, Hwang M-J. Detection and classification of cardiac arrhythmias by a challenge-best deep learning neural network model. Iscience. 2020;23(3). 10.1016/j.isci.2020.100886 PMC7031313 32062420

[6] Huang J-S, Chen B-Q, Zeng N-Y, Cao X-C, Li Y. Accurate classification of ECG arrhythmia using MOWPT enhanced fast compression deep learning networks. Journal of Ambient Intelligence and Humanized Computing. 2020, pages 1–18.

[7] Huang Z, Herbozo Contreras LF, Yu L et al. S4D-ECG: A shallow state-of-the-art model for cardiac abnormality classification. Cardiovasc Eng Tech 2024. 10.1007/s13239-024-00716-3 PMC11239723 38332408

[8] Hasani R, Lechner M, Amini A, Rus D, Grosu R. Liquid time-constant networks. In: Proceedings of the AAAI Conference on Artificial Intelligence, volume 35, pages 7657–7666, 2021.

[9] Hasani R, Lechner M, Amini A, Liebenwein L, Ray A, Tschaikowski M, Teschl G, Rus D. Closed-form continuous-time neural networks. Nat Mach Intell. 2022;4(11):992–1003. 10.1038/s42256-022-00556-7

[10] Liu F, Liu C, Zhao L, Zhang X, Wu X, Xu X, Liu Y, Ma C, Wei S, He Z, et al. An open access database for evaluating the algorithms of electrocardiogram rhythm and morphology abnormality detection. J Med Imaging Health Inform. 2018;8(7):1368–73. 10.1166/jmihi.2018.2442

[11] Ribeiro AH, Ribeiro MH, Paixão GMM, Oliveira DM, Gomes PR, Canazart JA, Ferreira MPS, Andersson CR, Macfarlane PW, Meira W Jr, et al. Automatic diagnosis of the 12-lead ECG using a deep neural network. Nat Commun. 2020;11(1):1760. 32273514 10.1038/s41467-020-15432-4 PMC7145824

[12] Wang Z, Chen Z, Wang X, Zhang L, Li S, Tian Y, Shao L, Hu H, Gao R, et al. The disease burden of atrial fibrillation in China from a national cross-sectional survey. Am J Cardiol. 2018;122(5):793–8. 30049467 10.1016/j.amjcard.2018.05.015

[13] Chiu SN, Lin LY, Wang JK, Lu CW, Chang CW, Lin MT, Hua YC, Lue HC, Wu MH. Long-term outcomes of pediatric sinus bradycardia. J Pediatr. 2013;163(3):885–9. 23623512 10.1016/j.jpeds.2013.03.054

[14] Still A-M, Raatikainen P, Ylitalo A, Kauma H, Ikäheimo M, Kesäniemi YA, Huikuri HV. Prevalence, characteristics and natural course of inappropriate sinus tachycardia. EP Europace. 2005;7(2):104–12. 15763524 10.1016/j.eupc.2004.12.007

[15] Xiong Y, Wang L, Liu W, Hankey GJ, Xu B, Wang S. The prognostic significance of right bundle branch block: a meta-analysis of prospective cohort studies. Clin Cardiol. 2015;38(10):604–13. 26436874 10.1002/clc.22454 PMC6490823

[16] Pérez-Riera AR, Barbosa-Barros R, de Rezende Barbosa MPC, Daminello-Raimundo R, de Abreu LC, Nikus K. Left bundle branch block: epidemiology, etiology, anatomic features, electrovectorcardiography, and classification proposal. Ann Noninvasive Electrocardiol. 2019;24(2). 10.1111/anec.12572 PMC6931474 29932265

[17] Nikolaidou T, Ghosh JM, Clark AL. Outcomes related to first-degree atrioventricular block and therapeutic implications in patients with heart failure. JACC: Clinical Electrophysiology. 2016;2(2):181–92. 29766868 10.1016/j.jacep.2016.02.012

[18] Huang Z, MacLachlan S, Yu L, Herbozo Contreras LF, Truong ND, Ribeiro AH, Kavehei O. Generalization challenges in ECG deep learning: insights from dataset characteristics and attention mechanism. medRxiv. 2023b;2023–07. 10.1080/14796678.2024.2354082 PMC11285255 39049767

[19] Butterworth S, et al. On the theory of filter amplifiers. Wireless Engineer. 1930;7(6):536–41.

[20] Tereshchenko LG, Josephson ME. Frequency content and characteristics of ventricular conduction. J Electrocardiol. 2015;48(6):933–7. 26364232 10.1016/j.jelectrocard.2015.08.034 PMC4624499

[21] Lin J, Chen W-M, Lin Y, Gan C. Han S. Mcunet: Tiny deep learning on IoT devices. Adv Neural Inf Process Syst; 2020. p. 33.

[22] Lin J, Chen W-M, Cai H, Gan C, Han S. MCUNetV2: memory-efficient patch-based inference for tiny deep learning. In: Annual Conference on Neural Information Processing Systems (NeurIPS). 2021.

[23] Mukhopadhyay S, Dey S, Ghose A, Tyagi A. Automated generation of tiny model for real-time ECG classification on tiny edge devices. Proceedings of the 20th ACM Conference on Embedded Networked Sensor Systems. 2022;756–757.

[24] Chen J, Jiang M, Zhang X, da Silva DS, de Albuquerque VHC, Wu W. Implementing ultra-lightweight co-inference model in ubiquitous edge device for atrial fibrillation detection. Expert Syst Appl. 2023;216: 119407. 10.1016/j.eswa.2022.119407

